# Improving Precipitation in Cryogenic Rolling 6016 Aluminum Alloys during Aging Treatment

**DOI:** 10.3390/ma16093336

**Published:** 2023-04-24

**Authors:** Xucheng Wang, Yu Liu, Yuanchun Huang

**Affiliations:** 1 College of Mechanical and Electrical Engineering, Central South University, Changsha 410083, China; 2 State Key Laboratory of High Performance Complex Manufacturing, Central South University, Changsha 410083, China; 3 Light Alloys Research Institute, Central South University, Changsha 410083, China

**Keywords:** AA6016 plate, cryogenic rolling, aging treatment, substructures, precipitation strengthening

## Abstract

This study systematically investigated the performance and microstructure characterization of cryogenic rolling (CR) and room-temperature rolling (RTR) Al–Mg–Si alloys. The result showed that the hardness of the CR alloys decreased at the early aging stage, but that the hardness of the RTR alloys increased at the early aging stage. Retrogression phenomena were apparent in the CR alloys at the early aging stage. Despite undergoing the same solid solution treatment, a few substructures were still observed in the CR alloys, and the degree of recrystallization in the CR alloys was significantly inferior to that in the RTR alloys. After aging for 50 h, the strength and precipitates’ density in the CR 75 alloy were higher than that in the other alloys; this indicated that the substructures were beneficial to precipitation and precipitate growth. A precipitation strength model was employed to illustrate the precipitation contribution at different aging stages. The results showed that the CR 75 alloy obtained the strongest precipitation strengthening.

## 1. Introduction

Heat-treatable Al–Mg–Si (6xxx) alloys have been widely used in the automotive industry due to their moderate strength, good formability and light weight [1,2,3]. With further economic growth, the pursuit of outstanding alloy strength has become the focus of the aluminum industry. Numerous methods have been developed to improve the mechanical properties of Al–Mg–Si alloys, such as equal-channel angular pressing (ECAP) [4,5], high-pressure torsion (HPT) [6,7], accumulative roll bonding (ARB) [8,9], friction stir processing [10] and cryogenic rolling (CR) [11,12]. Among these methods, cryogenic rolling has been the most widely accepted for the production of bulk ultrafine-grained (UFG) materials. Compared to room-temperature rolling (RTR), superior mechanical properties can be obtained by cryogenic rolling—this is due to the suppression of recovery and the reservation of dislocations during cryogenic rolling [11].

Although cryogenic rolling can significantly improve materials’ properties, it is widely accepted that precipitation strengthening caused by aging treatments still plays an important role in the strength of Al–Mg–Si alloys. The contribution of precipitation strengthening mainly results from the interaction between nanoscale precipitates and dislocations [13,14]. Various cryogenic rolling processes can result in diverse dislocation densities and dislocation structures that prominently affect precipitation. Researchers have shown that high dislocation densities are apt to reduce the supersaturation of matrices to a level that promotes nucleation, but that the attraction of solutes to dislocations could decrease the driving force for nucleation [15,16]. The precipitation sequence in Al–Mg–Si alloys is generally considered as [17,18,19]: super-saturated solid solution (SSSS) → atomic clusters → GP zones → *β*″→ *β*′, *U1*, *U2*, B′ → *β*, Si. A Mg_2_Si phase (i.e., *β* phase) and *β*″ phase are widely deemed as efficient strengthening precipitates in Al–Mg–Si alloys [14,20,21]. Therefore, understanding information about precipitates during aging treatments is vital in Al–Mg–Si alloys.

Recently, many experts have focused on the interactions between dislocations and precipitates in Al–Mg–Si alloys [14,20,22]. Dislocations are considered to shear the *β*″ phase [22,23] but bypass the *β*′ phase [23,24]. In an early study [25], the critical shear radius was used as a vital parameter to establish a yield strength model that included inherent strengthening, solution strengthening and precipitation strengthening; this model has been accepted widely in aging treatments. In recent years, other, similar models have also included the influence of microstructural characteristics [26,27,28]. Hitherto, the precipitation behaviors in Al–Mg–Si alloys have mainly focused on room-temperature deformation and hot deformation [29,30,31]. In order to greatly improve alloy strength, researchers have mainly focused on the process of “solution treatment + CR + aging treatment” [32,33,34], and this process improves alloys strength significantly. Superior mechanical properties obtained by low-temperature deformation in Al alloys have been attributed to the complicated effects of multiple strength mechanisms. For 6016 Al alloys, their main application is in manufacturing high-strength Al alloy automotive plates [3,17]. Although the process of “solution treatment + CR + aging treatment” can increase an alloy’s strength, the anisotropy brought on by its texture is difficult to reduce in subsequent aging processes. Krishna’s research [12] has shown that cryorolled alloys show an enhanced texture index value and a high IPA value, exhibiting enhanced anisotropic behavior compared to RTR-rolled alloys. Higher anisotropy could be harmful to subsequent manufacture processes [1].

Therefore, it is vital to explore processes that can not only improve the alloy’s strength, but also reduce the anisotropy caused by the deformation texture; hence, a process of “CR + solution treatment + aging treatment” was designed. Precipitation strengthening is vital after subsequent aging treatments. When CR alloys undergo solution treatments, partial recrystallization can reduce the anisotropy caused by their texture. 

In the present work, the aim was to illustrate the superior mechanical properties of CR alloys after aging treatments, to reduce the anisotropy caused by deformation texture and to employ the precipitation model to discuss precipitation strength during aging treatments.

## 2. Experimental and Theoretical Methods

### 2.1. Experimental Methods

The materials were twin-cast rolled 6016 alloy plates (4 mm thickness) that had been annealed. The composition of the 6016 alloy is shown in Table 1.

**Figure 1:**
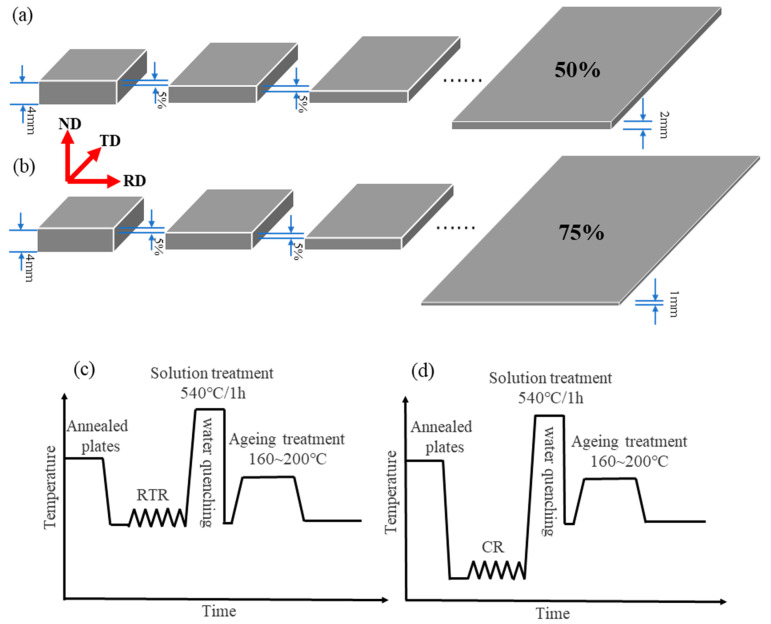
The rolling process of the 6016 aluminum alloy at (**a**) 50 % and (**b**) 75%; The process diagram for the (**c**) RTR and (**d**) CR.

One part of the plates was rolled up to a 50% and 75% reduction in thickness at liquid nitrogen temperature (hereafter labeled CR 50 and CR 75). The rolling process was carried out at 0.05 m/s with a 5% reduction in thickness per pass, as shown in Figure 1a,b. For comparison, another part of the plates was rolled with the same thickness reductions at room temperature (hereafter labeled RTR 50 and RTR 75). The CR alloys were immersed in a liquid nitrogen bath (−195 °C) for 30 min before rolling and were immersed in the liquid nitrogen for 2 min after every intermediate rolling pass. The rolled plates were solution heat-treated at 540 °C for 1 h and quenched in water at room temperature; all alloys were subsequently artificially aged at 160–200 °C for various periods of time. A schematic illustration of the whole process is shown in Figure 1c,d.

The microhardness of the alloys was tested with a load of 100 g and a dwell time of 15 s, excluding the maximum and minimum values. Quantitative X-ray diffraction (XRD, Cu Kα radiation, scan rate: 5°/min, scan step size: 0.02°, scan angle range: 30°–80°) measurements were performed with a D/max 2550 VB. Transmission electron microscopy (TEM) specimens were polished to a thickness of 50–80 μm and then punched into a Φ3 mm disc. The foil was subjected to twin-jet electropolishing with a mixed solution of 30% nitric acid and 70% methanol at −25 °C, at a subsequent voltage of 20 V. TEM observations were conducted on a Tecnai G2 F20 S-TWIN TMP operated with an accelerating voltage of 200 kV and a variable operating current in the range of 104–108 mA. Electron back-scattered diffraction samples were taken from the ND-RD section of the plate, and the microstructure was characterized by a Sirion 200 field emission scanning electron microscope (FEI Corporation, Valley City, ND, USA). The Electron Back-Scattered Diffraction (EBSD) samples were mechanically ground, followed by electro-polishing in an ethanol–perchloric acid solution (ratio of ethanol to perchloric acid of 9:1) at a temperature of −20 °C and a voltage of 20 V. The EBSD data were analyzed using HKL Channel 5 software.

### 2.2. Theoretical Methods

Researchers have established a strong obstacle model for the peak aging stage and a weak obstacle model for the under aging stage [35]. This model concluded that the critical resolved shear stress is determined from the interaction of gliding dislocations with point obstacles, while the average obstacle spacing is estimated by their shapes and orientation relationships with the matrix [25,35]. The strong obstacles model is given by Equation (1):

(1) $$\sigma_{ppt}=\frac{MF_{peak}f_{peak}^{1/2}}{br_{peak}{\left(2\pi \right)}^{1/2}}f_{r}^{1/2}$$

where *M* is the Taylor factor, *b* is the magnitude of the Burgers vector and *r_peak_* and *F_peak_* represent the average radius cross-section and the average obstacle strength of the precipitates at the peak aging stage, respectively. *f_peak_* is the volume fraction of the precipitates at the peak aging stage—this is a constant parameter [25]—and *f_r_* is the relative volume fraction of the precipitates, which is defined as in Equation (2):

(2) $$f_{r}=\frac{f}{f_{peak}}$$

where *f* is the volume fraction of the precipitates, and is calculated as follows:

(3) $$f=\frac{4}{3}\pi r_{eq}{}^{3}N$$

where *N* and *r_eq_* represent the precipitate number density and the precipitate equivalent radius, respectively. *N* and *r_eq_* are defined as follows:

(4) $$N=\frac{3N_{cs}}{At}$$

(5) $$r_{eq}={\left(\prod_{1}^{n}r_{n}\right)}^{1/n}$$

where *N_cs_* is the number of precipitate cross-sections in the image, *A* is the field of view area, *t* is thickness in the center of the image given in Ref. [28], *r_n_* is the radius of the measured precipitate radius and *n* is the number of measured precipitates. The weak obstacles model is expressed as Equation (6):

(6) $$\sigma_{ppt}=\frac{MF_{peak}^{3/2}f_{peak}^{1/2}}{b{\left(2\sqrt{3}\pi \right)}^{1/2}\Gamma^{1/2}r_{peak}^{3/2}}r^{1/2}f_{r}^{1/2}$$

where Г represents the dislocation line tension—described as Г *= Gb*^2^/2—*r* = (3/2)^1/4^*r_acs_* is the radius of an average equivalent circular and *r_acs_* is the average cross-section radius of the precipitates [36]. 

*σ_dis_* is usually assumed to scale with the dislocation density shown as Equation (7) [15,25]:

(7) $$\sigma_{dis}=M\alpha Gb\sqrt{\rho }$$

where *b* is the Burgers vector, *G* is the shear modulus, *α* is a geometric constant, *M* is the Taylor factor and *ρ* represents dislocation density, which can be given by Equation (8) [37]:

(8) $$\frac{\beta \cos\theta }{\lambda }=\frac{1}{D_{v}}+\frac{4\varepsilon \sin\theta }{\lambda }$$

where *β* is integral breadth, *λ* is the wavelength, *D_v_* is the volume-weighted average crystallite size and *ε* is the micro-strain. The slope and intercept of *β*cos*θ* with 4 sin*θ* represent the micro-strain value (*ε*) and the volume-weighted average crystallite size (*D_v_*), respectively. The basic assumption of the Williamson–Hall technique is that both the size and strain-broadened profiles are of a Lorentzian shape [38]. The dislocation density can be given by Equation (9) [38,39]:

(9) $$\rho =\rho_{d}{}^{1/2}\times \rho_{s}{}^{1/2}$$

where *ρ_d_* is the dislocation density due to the domain size, and *ρ_s_* is the dislocation density due to strain broadening. These are calculated as in Equations (10) and (11):

(10) $$\rho_{d}=\frac{3}{D_{v}^{2}}$$

(11) $$\rho_{s}=\frac{K\varepsilon^{2}}{b^{2}}$$

where *K* = 6π and *b* is the Burgers vector for the FCC structure.

## 3. Results

### 3.1. Mechanical Properties

**Figure 2:**
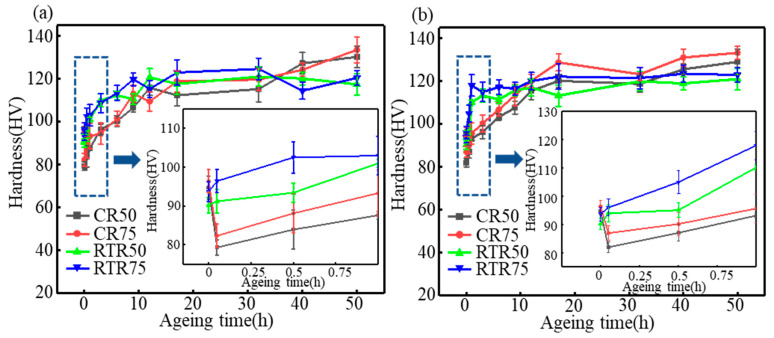

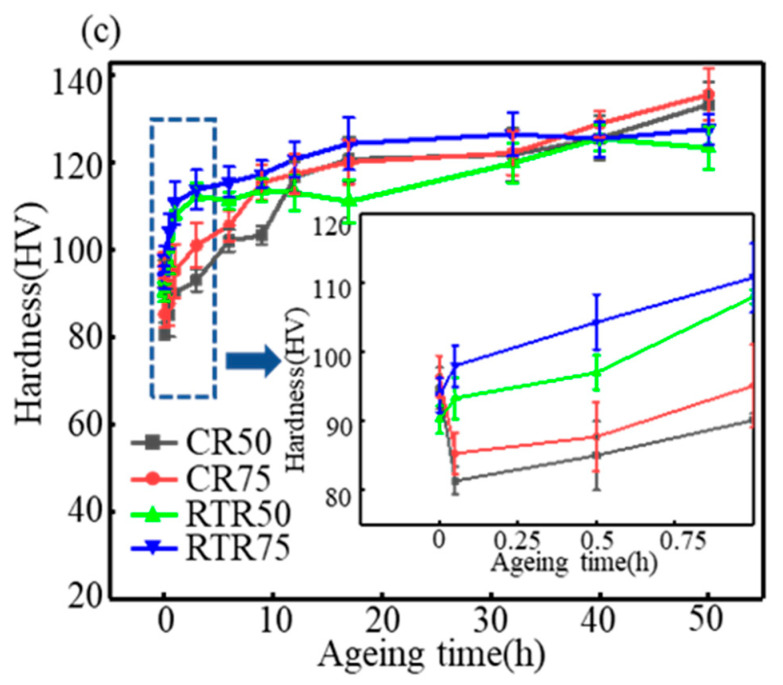
Age-hardening curves of the CR and RTR alloys at (**a**) 160 °C, (**b**) 180 °C and (**c**) 200 °C.

Figure 2a–c shows the age-hardening curves of the CR alloys and the RTR alloys at various temperatures. Compared to the CR alloys, the RTR alloys reached peak hardness in less time. The hardness of the RTR alloys barely increased when the samples were aged at 160 °C after 20 h; this indicates that the hardness of the RTR alloys nearly reached the peak, but the hardness of the CR alloys still increased when the samples were aged at 160 °C after 20 h. However, the age-hardening capacities (i.e., the increase in hardness during the aging treatment) of the RTR alloys were significantly inferior to those of the CR alloys; this indicates that a prolonged aging time made the CR alloys reach a superior hardness—the hardness of the CR alloys continued to increase when the alloys were aged for 40–50 h. 

However, a different phenomenon appeared between the CR alloys and RTR alloys regardless of the aging temperature: the hardness of the CR alloys first decreased in a short amount of time and then increased rapidly, but the hardness of the RTR alloys continuously increased as the aging time increased. Similar phenomena have appeared in many research works [8,17,40], and the results showed that the hardness of the different samples did not increase rapidly at the early stage of artificial aging. As the samples inevitably underwent natural aging (NA) between the quenching and artificial aging, the CR alloys could be more sensitive to NA. The early stage of the artificial aging caused the retrogression phenomenon and reduced the hardness of the CR alloys.

### 3.2. X-ray Diffraction Analysis

**Figure 3:**
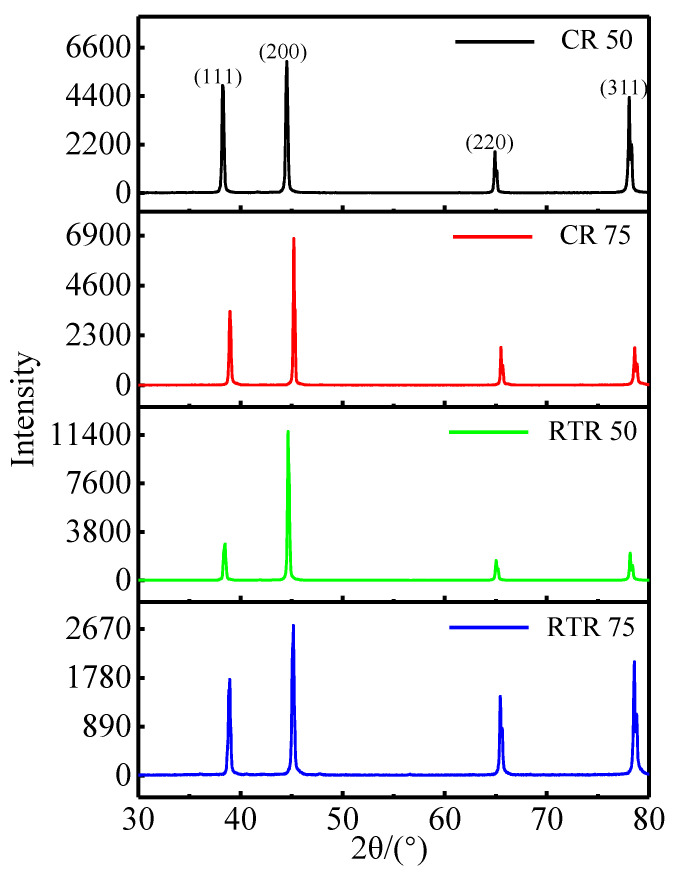
XRD patterns of the samples after CR and RTR, before the solution treatment.

Figure 3 shows the XRD patterns of the CR and RTR alloys before the solution treatment. It was significant that the intensity of the orientation along the (200) crystal plane was mostly higher than that of the other orientations; this was due to the severe accumulation of strain in the rolling direction [41]. According to the Williamson–Hall technique [37], the crystallite size, micro-strain and dislocation density are shown in Table 2. The crystallite sizes in the CR alloys were smaller than those in the RTR alloys, but the micro-strains in the CR alloys were higher than those in the RTR alloys. Large plastic deformations generated high dislocation densities. The dislocation densities of the 50%-reduction alloys were inferior to those of the 75%-reduction alloys. Meanwhile, the dislocation densities in the CR alloys were significantly higher than those in the RTR alloys; this shows that CR improved the dislocation density effectively, and that the deformation energy storage in the CR alloys was higher than that in the RTR alloys. This had a significant influence on the subsequent heat treatment process.

### 3.3. TEM Characterization

**Figure 4:**
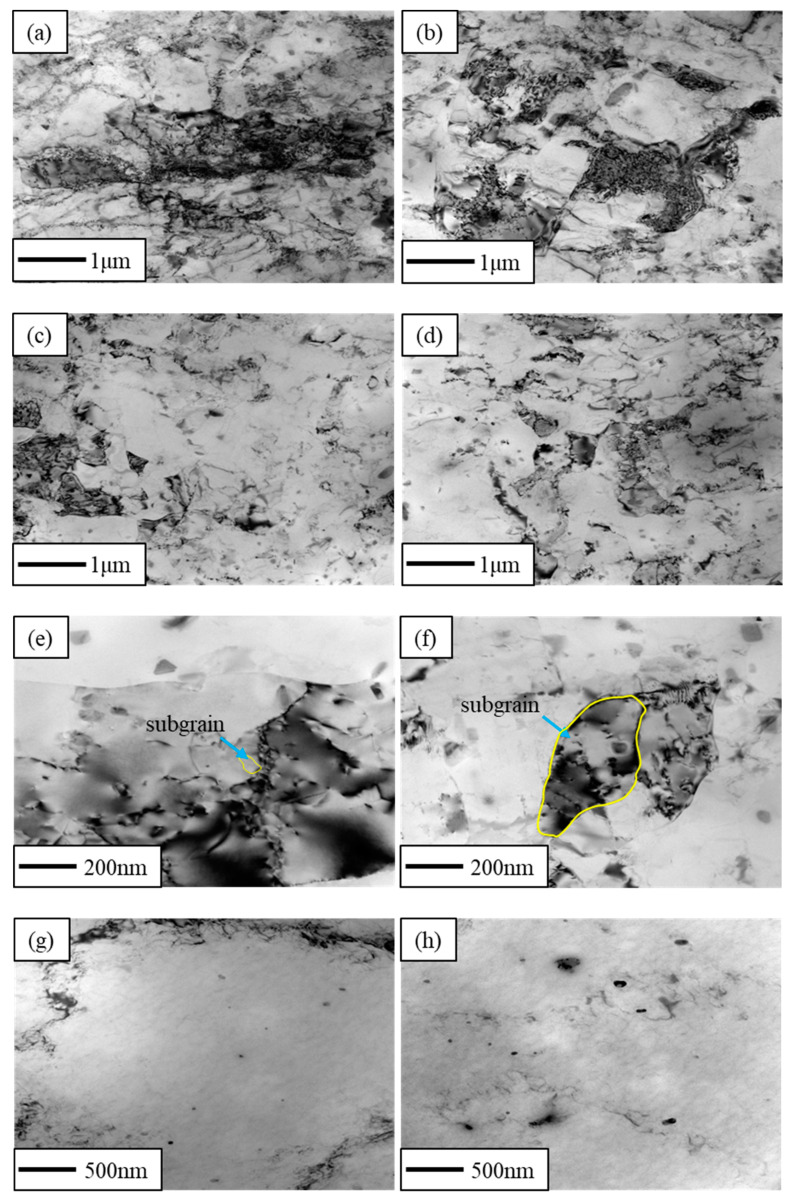
TEM images of alloys before solution treatment: (**a**) CR 75, (**b**) CR 50, (**c**) RTR 75 and (**d**) RTR 50; TEM images of alloys after solution treatment: (**e**) CR 75, (**f**) CR 50, (**g**) RTR 75 and (**h**) RTR 50.

Figure 4a–d shows the TEM bright field of the CR and RTR alloys before the solution treatment. It is significant that many dislocations were found in both the RTR and CR samples, and that the dislocation densities in the CR alloys were higher than those in the RTR alloys. Figure 4e,f shows the TEM bright field of the CR and RTR alloys after solution treatment. Subgrains were still observed in the CR alloys, and dislocations surrounded these subgrains. The subgrain size in the CR 75 alloy was less than that of the CR 50 alloy. However, few dislocation structures were found in the RTR alloys after the solution treatment.

**Figure 5:**
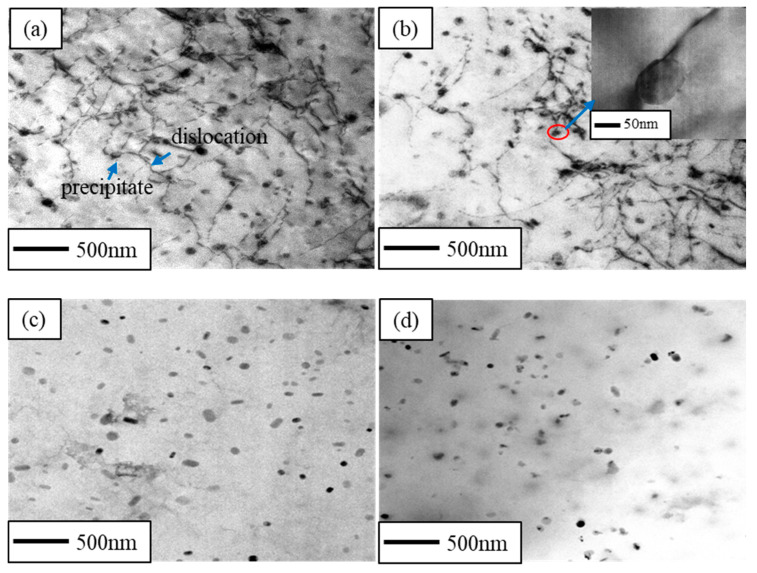
TEM images of alloys aged at 160 °C for 3 min: (**a**) CR 75, (**b**) CR 50, (**c**) RTR 75 and (**d**) RTR 50.

Figure 5a,b shows the TEM bright field of the CR alloys after 3 min of the aging treatment. After 3 min of the aging treatment, there were still a few dislocation structures in the CR alloys, and globular precipitates precipitated near these dislocations to prevent the dislocation from slipping; this indicates that dislocations in the CR alloys did not entirely disappear, although they underwent a short aging treatment. Nevertheless, in the RTR alloys (Figure 5c,d), dislocations were not found—only globular precipitates precipitated in the Al alloys. A previous study [42] confirmed that a large amount of globular Mg_2_Si phase is precipitated in Al–Mg–Si alloys, achieving an ultimate tensile strength of 421 MPa—which is greatly improved compared to the conventional T6 state. 

**Figure 6:**
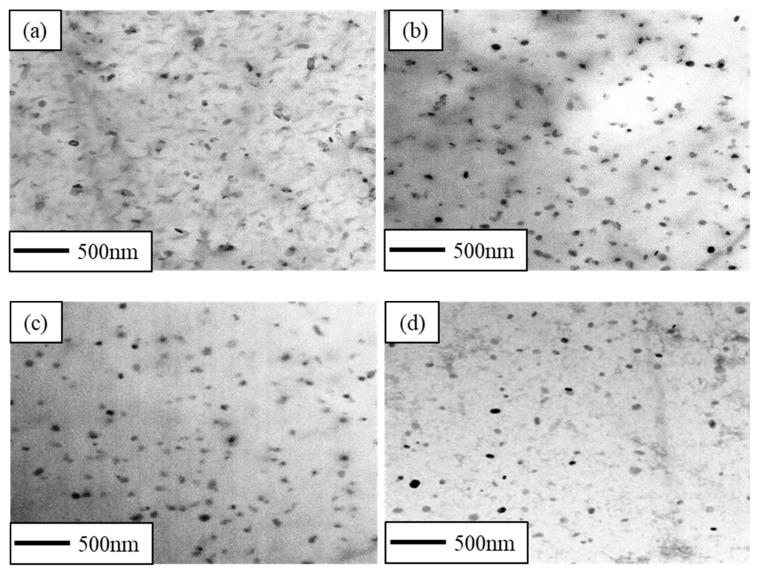
TEM images of alloys aged at 160 °C for 50 h: (**a**) CR 75, (**b**) CR 50, (**c**) RTR 75 and (**d**) RTR 50.

**Figure 7:**
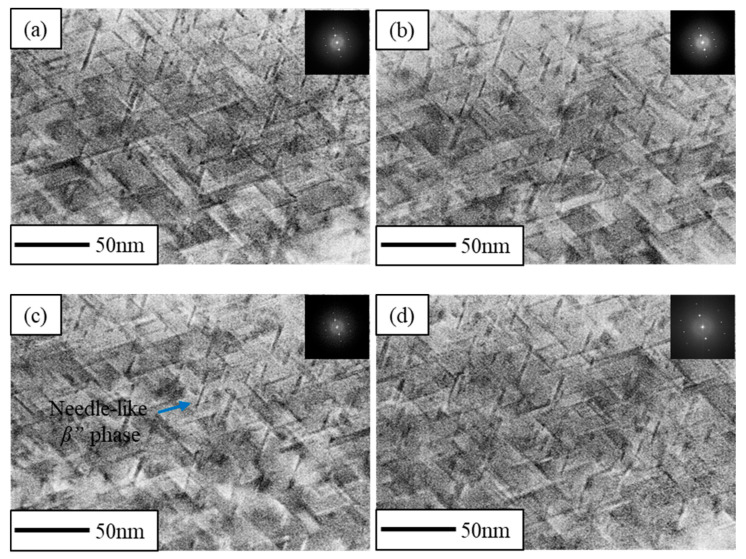
Needle-like *β*″ phase precipitated in different alloys after 160 °C × 50 h aging treatment: (**a**) CR 75, (**b**) CR 50, (**c**) RTR 75 and (**d**) RTR 50.

Figure 6a–d shows the TEM bright field of the CR and RTR alloys after 50 h of the aging treatment. It was apparent that a mass of globular Mg_2_Si phases precipitated in both the CR and RTR alloys. The density of the Mg_2_Si phase in the CR 75 alloy was the highest, and the density of the Mg_2_Si phase in the RTR 50 alloy was the lowest. The Mg_2_Si phase was the main strengthening phase in the 6xxx Al alloy, so the densities of the Mg_2_Si phases affected the precipitation strengthening significantly; this indicates that the precipitation strengthening in the CR 75 alloy was superior to other alloys. Meanwhile, the needle-like *β*″ phase is widely considered another strengthening phase in Al–Mg–Si alloys [14,17,20]. These needle-like *β*″ phases were found in both of the CR and RTR alloys after 50 h of the aging treatment. However, the density of the needle-like *β*″ phase had no significant difference, as shown in Figure 7a–d.

### 3.4. EBSD Characterization

**Figure 8:**
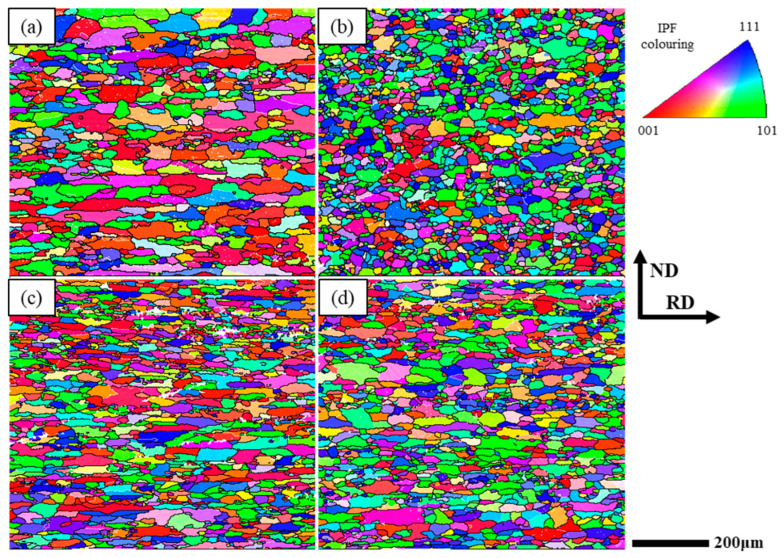

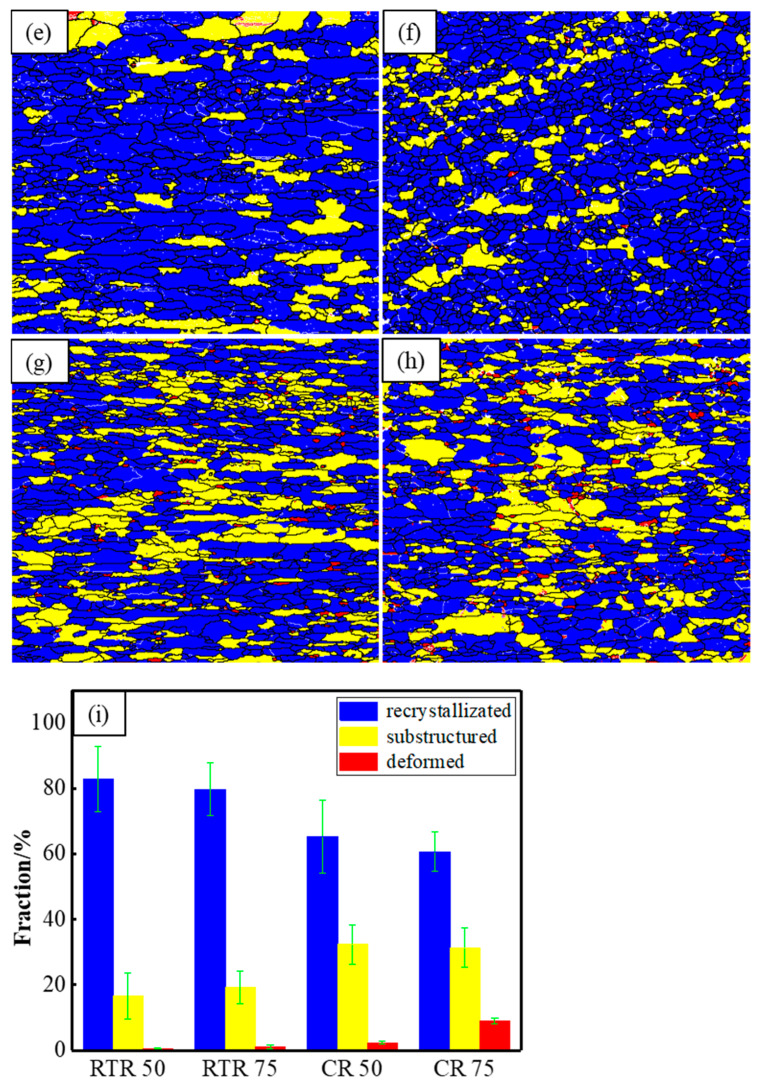
IPF after solution treatment: (**a**) RTR 50, (**b**) RTR 75, (**c**) CR 50 and (**d**) CR 75; degree of recrystallization and recovery: (**e**) RTR 50, (**f**) RTR 75, (**g**) CR 50 and (**h**) CR 75; (**i**) volume fraction.

**Figure 9:**
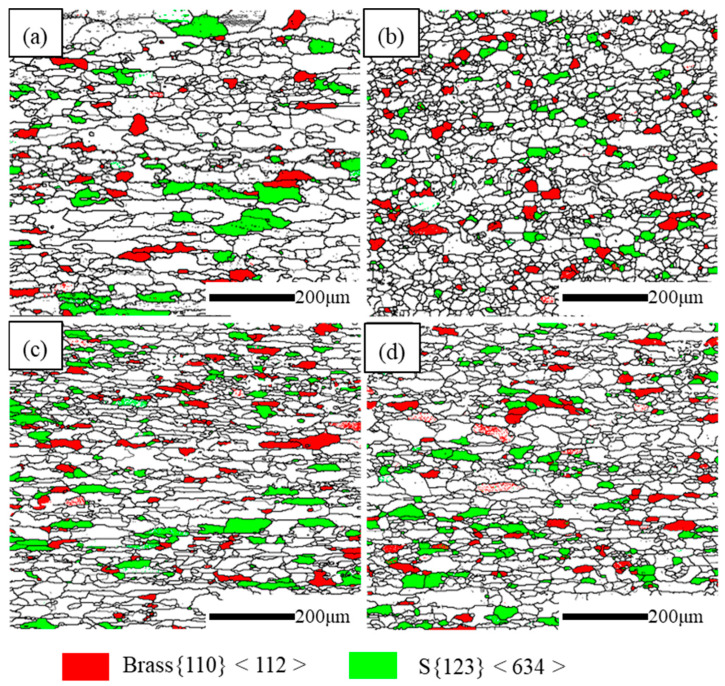
Deformation texture in different alloys: (**a**) RTR 50, (**b**) RTR 75, (**c**) CR 50 and (**d**) CR 75.

Figure 8a–d shows the IPF of the CR and RTR alloys. It is significant that the average grain size of the RTR 75 alloy was the finest. The average grain sizes of the RTR alloys were finer than those of the CR alloys. The degree of recrystallization and recovery in the CR and RTR alloys is shown in Figure 8e–i. Despite the solid solution treatment, the CR and RTR alloys did not fully recrystallize; there were a few substructures and deformed structures in the alloys. The statistical results show that the degree of recrystallization in the CR alloy was inferior to that in the RTR alloy, but the degree of recovery in the CR alloy was superior to that in the RTR alloy. Substructures (such as dislocation) remained in the CR alloys, which indicated that the CR alloy was prone to recovery during the solution treatment. The recrystallization and the substructure in the RTR 50 alloy were similar to those in the RTR 75 alloy; few deformation structures existed in the RTR alloys. The recrystallization and the substructures in the CR 50 alloy were also similar to those in the CR 75 alloy. However, the deformed structures in the CR 50 alloy were inferior to those in the CR 75 alloy indicating that more deformation energy existed in the CR 75 alloy. Figure 9a–d shows the deformation texture in the different alloys, and the statistical results are shown in Table 3. After the solution treatment, a few deformation textures (Brass {110} <112> and S {123} <634>) still existed in both of the RTR and CR alloys; the proportion of deformation texture in the CR alloys was similar to that in the RTR alloys. Compared with the alloys in Ref. [12], the proportion of deformation texture in the CR alloys was reduced significantly; this resulted in the anisotropy caused by the deformation texture decreasing significantly.

## 4. Discussion

### 4.1. Microstructure and Precipitate Evolution during Aging Treatment

Compared with the RTR alloys, dislocations in the CR alloys were significantly higher than those in the RTR alloys—as shown in Figure 4a–d. As shown in Figure 4e–h, despite the solution treatment, subgrains were observed in the CR alloys, while these subgrains were not observed in RTR alloys. The hardness variations in the CR and RTR alloys were opposite at the early aging stage: the hardness of the CR alloys decreased, but the hardness of the RTR alloys increased. Researchers [17] found that the hardness, yield strength and tensile strength of the natural aging AA6016 aluminum alloys decreased first, and then increased when the alloys were artificially aged at 185°C. This phenomenon was attributed to the dissolution of clusters formed during natural aging (NA), caused by retrogression and re-aging. A. Serizawa et al. [43] pointed out that the water-quenched alloys were able to form a kind of atomic cluster when alloys were naturally aged; the density gradually increased at the natural aging stage. As a result, supersaturated solute atoms and vacancies in the alloy matrix were consumed in large quantities, and the nucleation growth of the precipitate was delayed. Compared with the RTR alloys, the CR alloys tended to form clusters more easily during NA, due to the substructures formed after the solution treatment (Figure 4e,f). 

Grains were broken and elongated during cryogenic rolling, and the work-hardening phenomenon was evident due to the presence of a lot of dislocation tangles. As shown in Figure 8e–h, the proportion of recrystallization in the CR alloys was inferior to that in the RTR alloys. Since the recovery of the CR alloy was inhibited during the rolling process, more dislocations were retained. These irregular dislocations were distributed on different slip planes due to the multiple slip systems in the Al alloy. It was difficult for the Al alloy to recrystallize because of its high layer fault energy; the solution treatment made the edge dislocation climb more easily, which resulted in these dislocation tangles being redistributed on different slip planes to form substructures rather than recrystallization. After the solution treatment, more substructures existed in the CR alloys than in the RTR alloys. The substructures and deformed structures in the CR 75 alloy were higher than those in the RTR alloys, which indicated that there were more substructures in the CR 75 alloy to provide nucleation for precipitation during the aging treatment. The substructures partly remained after the solution treatment in the CR alloys, which resulted in better substructure strengthening (Figure 4e,f). As shown in Figure 5b, the plate-like Mg_2_Si phase was pinned at the dislocation and hindered the dislocation from moving. Supersaturation, substructures and precipitates existed in the CR alloys at the early aging stage. As aging time increased, this supersaturation and the substructures gradually decreased, and the density of precipitates gradually increased; precipitation strengthening was greatly enhanced.

When the aging time reached 20 h, the hardness of both the CR and RTR alloys rose slowly—the reasons were concluded to be as follows:

1. The effect of solution strengthening on alloy strength was nearly exhausted due to the long-time aging treatment.
2. Precipitation strengthening increased slowly. As shown in Equation (12) [15] and Equation (13) [44], when the concentration of alloying elements in the solid solution (*C*) went down towards the solution equilibrium concentration (*C_e_*)—which resulted in nucleation rate (*j*) and growth rate(d*r*/d*t*) decline—this showed that precipitation had continued to exhaustion, and so precipitation strengthening slowed down.

(12) $$j=j_{0}\exp\left[-{\left(\frac{A_{0}}{RT}\right)}^{3}{\left(\frac{1}{\mathrm{In}(C/C_{e})}\right)}^{2}\right]\exp\left(-\frac{Q_{d}}{RT}\right)$$

(13) $$\frac{dr}{dt}=\frac{\bar{C}-C_{i}}{C_{p}-C_{i}}\frac{D}{r}$$

### 4.2. Precipitation Strengthening during Aging Treatment

As shown in Figure 5, there were a few dislocations in the CR alloys after aging for 3 min, Mg_2_Si phases precipitated in both the CR and RTR alloys. Compared with the RTR alloys, more plate-like Mg_2_Si phases precipitated in the CR alloys after aging for 50 h (Figure 6a–d)—in particular, the density in the CR 75 alloy was higher than in the other alloys, indicating that the precipitation strengthening of the CR alloy was higher than that in the RTR alloy. Mg_2_Si phase, as the main strengthening phase in Al–Mg–Si alloys, contributes to alloy strength significantly; this contribution is mainly reflected in the prevention of dislocation slipping. A high density of precipitates indicates a strong resistance to dislocations. The substructures retained in the CR alloys after the solution treatment not only brought substructural strengthening, but also benefited precipitation during the aging process. When aging time was increased to 50 h, the dislocations in the CR alloys nearly disappeared; the dislocation removal provided energy for precipitate nucleation and growth during aging treatment. The densities of needle-like *β*″ phases in the CR and RTR alloys were approximately similar after aging for 50 h (Figure 7a–d); this shows that the difference in strength between the CR and RTR alloys was not related to the *β*″ phase. 

### 4.3. The Effect of Deformation Texture after Solution Treatment

Texture variation plays an important role in the materials’ properties as its effect on anisotropy can be used to improve the formability of Al–Mg–Si autobody sheets [1]. In the “solution treatment + CR + aging treatment” process, the CR sample showed a 27.99% Brass texture and a 26.72% S texture; the anisotropy behavior of the CR sample was enhanced compared with the RTR sample [12]. However, the deformation texture was reduced significantly in the “CR + solution treatment + aging treatment” process, as shown in Table 3. Although the “CR + solution treatment + aging treatment” process sacrificed the partial dislocation strengthening caused by cryorolling, it improved the alloy precipitation strength—reducing the proportion of deformation texture and the anisotropy of the plate.

### 4.4. The Contributions of Different Strength Mechanisms

**Figure 10:**
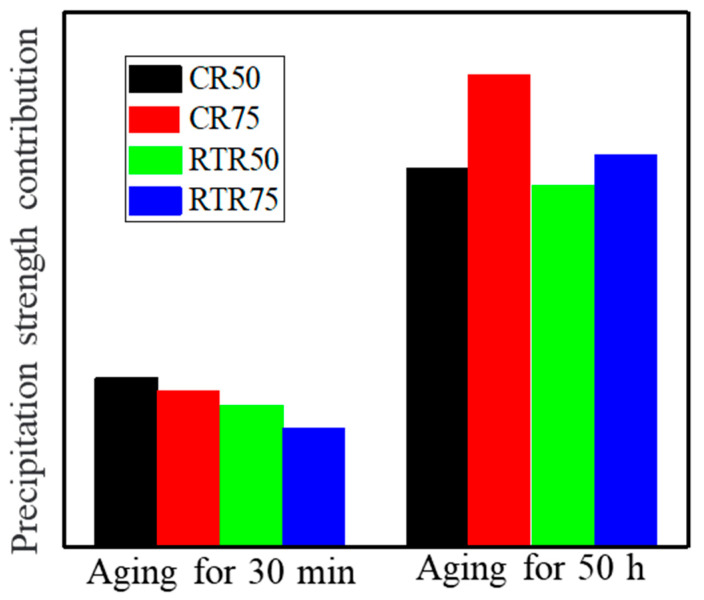
The contribution of precipitation strengthening when alloys were aged at 160 °C for 50 h.

Equations (1)–(6) were employed to reveal the precipitation strengthening contributions during the aging treatment, as shown in Figure 10. At the early aging stage, the precipitation strengthening contribution of the CR and RTR alloys showed little difference. However, when alloys were aged at 160 °C for 50 h, the precipitation strengthening contribution of the CR 75 alloy had significant advantages over the others; more substructures remained after the solution treatment in the CR 75 alloy. As aging time increased, these substructures gradually disappeared. The precipitation strengthening in the CR 75 alloy improved more significantly than others due to high precipitate densities (Figure 6a); this resulted in the aging hardening capacity of the CR 75 being superior to the other alloys (Figure 2a–c), and the CR 75 alloy had the highest strength (Figure 6d). 

## 5. Conclusions

The performance and microstructure characterization of CR and RTR alloys were investigated. Compared to RTR, more dislocations existed in alloys after CR due to the suppression of recovery. The hardness of the CR alloys decreased at the early aging stage, but the hardness of the RTR alloys increased at the early aging stage; this is because CR alloys could be more sensitive to NA, and so the retrogression phenomenon was apparent. The proportion of deformation textures was reduced after the solution treatment, which resulted in decreased anisotropy of the plate. After 50 h of the aging treatment, the hardness and the precipitate density of the CR 75 alloy were the highest; this indicates that the residual substructures after the solution treatment promoted precipitation and improved strength during the aging treatment. A precipitation strength model was employed to illustrate the precipitation contribution during the aging treatment; the results show that the CR 75 alloy obtained the strongest precipitation strengthening.

## Figures and Tables

**Table 1:** Chemical composition of cast-rolled 6016 aluminum.

Element	Si	Fe	Mn	Mg	Cr	Al
wt.%	1.48	0.34	0.16	0.49	0.1	Bal.

**Table 2:** Crystallite sizes, micro-strains and dislocation densities of samples.

Process Condition	*D_v_* (nm)	*ε* (×10^−3^)	*ρ* (m^−2^)
CR 50	177	0.29	2.06 × 10^17^
CR 75	105	0.33	3.91 × 10^17^
RTR 50	237	0.27	3.40 × 10^15^
RTR 75	223	0.31	2.93 × 10^15^

**Table 3:** Texture fraction of different samples.

Process Condition	Brass/%	S/%
RTR 50	8.3	8.2
RTR 75	10.1	10.1
CR 50	10.7	9.8
CR 75	12.5	11.2
LNR 75 [12]	27.99	26.72
RTR 75 [12]	20.96	23.06
